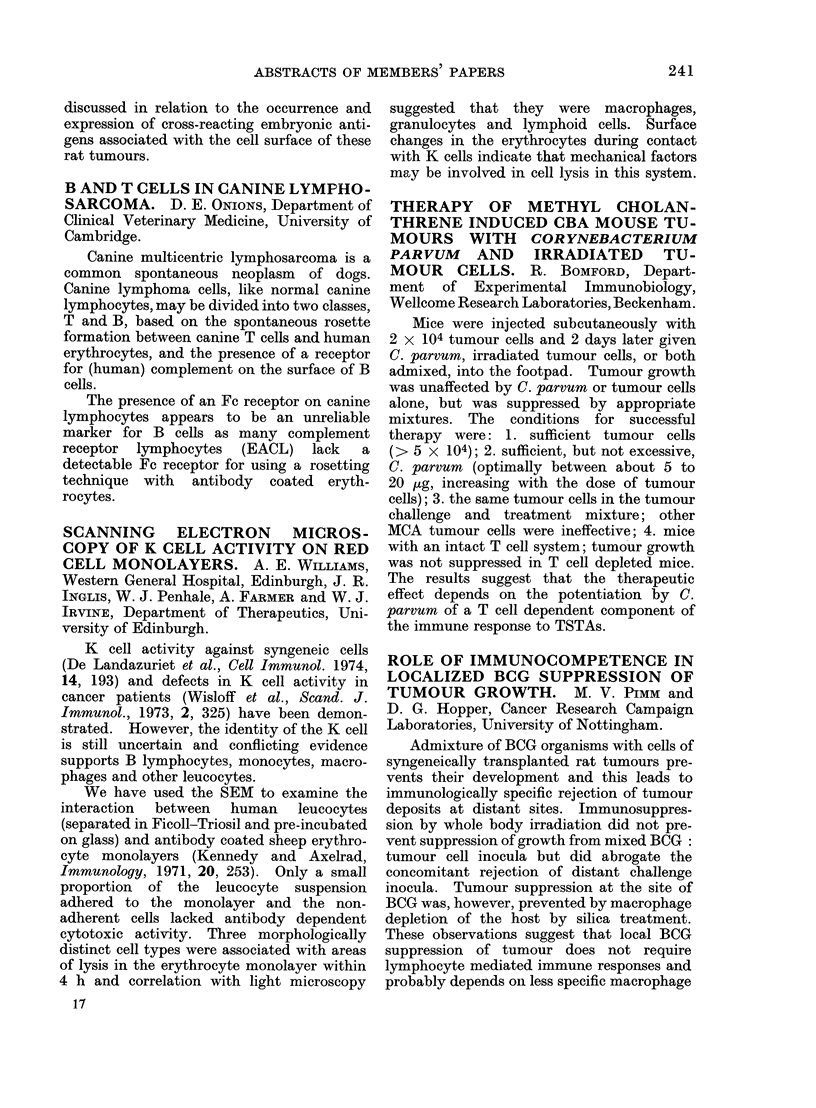# Proceedings: Therapy of methyl cholanthrene induced CBA mouse tumours with Corynebacterium parvum and irradiated tumour cells.

**DOI:** 10.1038/bjc.1975.159

**Published:** 1975-08

**Authors:** R. Bomford


**THERAPY OF METHYL CHOLANTHRENE INDUCED CBA MOUSE TUMOURS WITH *CORYNEBACTERIUM PARVUM* AND IRRADIATED TUMOUR CELLS.** R. Bomford, Department of Experimental Immunobiology, Wellcome Research Laboratories, Beckenham.

Mice were injected subcutaneously with $2 \times 10^4$ tumour cells and 2 days later given *C. parvum*, irradiated tumour cells, or both admixed, into the footpad. Tumour growth was unaffected by *C. parvum* or tumour cells alone, but was suppressed by appropriate mixtures. The conditions for successful therapy were: 1. sufficient tumour cells ($> 5 \times 10^4$); 2. sufficient, but not excessive, *C. parvum* (optimally between about 5 to 20 $\mu$g, increasing with the dose of tumour cells); 3. the same tumour cells in the tumour challenge and treatment mixture; other MCA tumour cells were ineffective; 4. mice with an intact T cell system; tumour growth was not suppressed in T cell depleted mice. The results suggest that the therapeutic effect depends on the potentiation by *C. parvum* of a T cell dependent component of the immune response to TSTAs.